# Evaluation of integrated community case management of the common childhood illness program in Gondar city, northwest Ethiopia: a case study evaluation design

**DOI:** 10.1186/s12887-024-04785-0

**Published:** 2024-05-09

**Authors:** Mekides Geta, Geta Asrade Alemayehu, Wubshet Debebe Negash, Tadele Biresaw Belachew, Chalie Tadie Tsehay, Getachew Teshale

**Affiliations:** 1 Metema District Health office, Gondar, Ethiopia; 2 Department of Health Systems and Policy, Institute of Public Health, College of Medicine and Health Sciences, University of Gondar, P.O. Box 196, Gondar, Ethiopia

**Keywords:** Integrated community case management, Evaluation, Gondar, Ethiopia

## Abstract

**Background:**

Integrated Community Case Management (ICCM) of common childhood illness is one of the global initiatives to reduce mortality among under-five children by two-thirds. It is also implemented in Ethiopia to improve community access and coverage of health services. However, as per our best knowledge the implementation status of integrated community case management in the study area is not well evaluated. Therefore, this study aimed to evaluate the implementation status of the integrated community case management program in Gondar City, Northwest Ethiopia.

**Methods:**

A single case study design with mixed methods was employed to evaluate the process of integrated community case management for common childhood illness in Gondar town from March 17 to April 17, 2022. The availability, compliance, and acceptability dimensions of the program implementation were evaluated using 49 indicators. In this evaluation, 484 mothers or caregivers participated in exit interviews; 230 records were reviewed, 21 key informants were interviewed; and 42 observations were included. To identify the predictor variables associated with acceptability, we used a multivariable logistic regression analysis. Statistically significant variables were identified based on the adjusted odds ratio (AOR) with a 95% confidence interval (CI) and p-value. The qualitative data was recorded, transcribed, and translated into English, and thematic analysis was carried out.

**Results:**

The overall implementation of integrated community case management was 81.5%, of which availability (84.2%), compliance (83.1%), and acceptability (75.3%) contributed. Some drugs and medical equipment, like Cotrimoxazole, vitamin K, a timer, and a resuscitation bag, were stocked out. Health care providers complained that lack of refreshment training and continuous supportive supervision was the common challenges that led to a skill gap for effective program delivery. Educational status (primary AOR = 0.27, 95% CI:0.11–0.52), secondary AOR = 0.16, 95% CI:0.07–0.39), and college and above AOR = 0.08, 95% CI:0.07–0.39), prescribed drug availability (AOR = 2.17, 95% CI:1.14–4.10), travel time to the to the ICCM site (AOR = 3.8, 95% CI:1.99–7.35), and waiting time (AOR = 2.80, 95% CI:1.16–6.79) were factors associated with the acceptability of the program by caregivers.

**Conclusion and recommendation:**

The overall implementation status of the integrated community case management program was judged as good. However, there were gaps observed in the assessment, classification, and treatment of diseases. Educational status, availability of the prescribed drugs, waiting time and travel time to integrated community case management sites were factors associated with the program acceptability. Continuous supportive supervision for health facilities, refreshment training for HEW’s to maximize compliance, construction clean water sources for HPs, and conducting longitudinal studies for the future are the forwarded recommendation.

## Background

Integrated Community Case Management (ICCM) is a critical public health strategy for expanding the coverage of quality child care services [1, 2]. It mainly concentrated on curative care and also on the diagnosis, treatment, and referral of children who are ill with infectious diseases [3, 4].

Based on the World Health Organization (WHO) and the United Nations Children’s Fund (UNICEF) recommendations, Ethiopia adopted and implemented a national policy supporting community-based treatment of common childhood illnesses like pneumonia, Diarrhea, uncomplicated malnutrition, malaria and other febrile illness and Amhara region was one the piloted regions in late 2010 [5]. The Ethiopian primary healthcare units, established at district levels include primary hospitals, health centers (HCs), and health posts (HPs). The HPs are run by Health Extension Workers (HEWs), and they have function of monitoring health programs and disease occurrence, providing health education, essential primary care services, and timely referrals to HCs [6, 7]. The Health Extension Program (HEP) uses task shifting and community ownership to provide essential health services at the first level using the health development army and a network of woman volunteers. These groups are organized to promote health and prevent diseases through community participation and empowerment by identifying the salient local bottlenecks which hinder vital maternal, neonatal, and child health service utilization [8, 9].

One of the key steps to enhance the clinical case of health extension staff is to encourage better growth and development among under-five children by health extension. Healthy family and neighborhood practices are also encouraged [10, 11]. The program also combines immunization, community-based feeding, vitamin A and de-worming with multiple preventive measures [12, 13]. Now a days rapidly scaling up of ICCM approach to efficiently manage the most common causes of morbidity and mortality of children under the age of five in an integrated manner at the community level is required [14, 15].

Over 5.3 million children are died at a global level in 2018 and most causes (75%) are preventable or treatable diseases such as pneumonia, malaria and diarrhea [16]. About 99% of the global burden of mortality and morbidity of under-five children which exists in developing countries are due to common childhood diseases such as pneumonia, diarrhea, malaria and malnutrition [17].

In 2013, the mortality rate of under-five children in Sub-Saharan Africa decreased to 86 deaths per 1000 live birth and estimated to be 25 per 1000live births by 2030. However, it is a huge figure and the trends are not sufficient to reach the target [18]. About half of global under-five deaths occurred in sub-Saharan Africa. And from the top 26 nations burdened with 80% of the world’s under-five deaths, 19 are in sub-Saharan Africa [19].

To alleviate the burden, the Ethiopian government tries to deliver basic child care services at the community level by trained health extension workers. The program improves the health of the children not only in Ethiopia but also in some African nations. Despite its proven benefits, the program implementation had several challenges, in particular, non-adherence to the national guidelines among health care workers [20]. Addressing those challenges could further improve the program performance. Present treatment levels in sub-Saharan Africa are unacceptably poor; only 39% of children receive proper diarrhea treatment, 13% of children with suspected pneumonia receive antibiotics, 13% of children with fever receive a finger/heel stick to screen for malaria [21].

To improve the program performance, program gaps should be identified through scientific evaluations and stakeholder involvement. This evaluation not only identify gaps but also forward recommendations for the observed gaps. Furthermore, the implementation status of ICCM of common childhood illnesses has not been evaluated in the study area yet. Therefore, this work aimed to evaluate the implementation status of integrated community case management program implementation in Gondar town, northwest Ethiopia. The findings may be used by policy makers, healthcare providers, funders and researchers.

## Method and material

### Evaluation design and settings

A single-case study design with concurrent mixed-methods evaluation was conducted in Gondar city, northwest Ethiopia, from March 17 to April 17, 2022. The evaluability assessment was done from December 15–30, 2021. Both qualitative and quantitative data were collected concurrently, analyzed separately, and integrated at the result interpretation phase.

The evaluation area, Gondar City, is located in northwest Ethiopia, 740 km from Addis Ababa, the capital city of the country. It has six sub-cities and thirty-six kebeles (25 urban and 11 rural). In 2019, the estimated total population of the town was 338,646, and 58,519 (17.3%) were under-five children. In the town there are eight public health centers and 14 health posts serving the population. All health posts provide ICCM service for more than 70,852 populations.

### Evaluation approach and dimensions

#### Program stakeholders

The evaluation followed a formative participatory approach by engaging the potential stakeholders in the program. Prior to the development of the proposal, an extensive discussion was held with the Gondar City Health Department to identify other key stakeholders in the program. Service providers at each health facility (HCs and HPs), caretakers of sick children, the Gondar City Health Office (GCHO), the Amhara Regional Health Bureau (ARHB), the Minister of Health (MoH), and NGOs (IFHP and Save the Children) were considered key stakeholders. During the Evaluability Assessment (EA), the stakeholders were involved in the development of evaluation questions, objectives, indicators, and judgment criteria of the evaluation.

### Evaluation dimensions

The availability and acceptability dimensions from the access framework [22] and compliance dimension from the fidelity framework [23] were used to evaluate the implementation of ICCM.

### Population and samplings

All under-five children and their caregivers attended at the HPs; program implementers (health extension workers, healthcare providers, healthcare managers, PHCU focal persons, MCH coordinators, and other stakeholders); and ICCM records and registries in the health posts of Gondar city administration were included in the evaluation. For quantitative data, the required sample size was proportionally allocated for each health post based on the number of cases served in the recent one month. But the qualitative sample size was determined by data saturation, and the samples were selected purposefully.

The data sources and sample size for the compliance dimension were all administrative records/reports and ICCM registration books (230 documents) in all health posts registered from December 1, 2021, to February 30, 2022 (three months retrospectively) included in the evaluation. The registries were assessed starting from the most recent registration number until the required sample size was obtained for each health post.

The sample size to measure the mothers’/caregivers’ acceptability towards ICCM was calculated by taking prevalence of caregivers’ satisfaction on ICCM program *p* = 74% from previously similar study [24] and considering standard error 4% at 95% CI and 10% non- responses, which gave 508. Except those who were seriously ill, all caregivers attending the ICCM sites during data collection were selected and interviewed consecutively.

The availability of required supplies, materials and human resources for the program were assessed in all 14HPs. The data collectors observed the health posts and collected required data by using a resources inventory checklist.

A total of 70 non-participatory patient-provider interactions were also observed. The observations were conducted per each health post and for health posts which have more than one health extension workers one of them were selected randomly. The observation findings were used to triangulate the findings obtained through other data collection techniques. Since people may act accordingly to the standards when they know they are observed for their activities, we discarded the first two observations from analysis. It is one of the strategies to minimize the Hawthorne effect of the study. Finally a total of 42 (3 in each HPs) observations were included in the analysis.

Twenty one key informants (14 HEWs, 3 PHCU focal person, 3 health center heads and one MCH coordinator) were interviewed. These key informants were selected since they are assumed to be best teachers in the program. Besides originally developed key informant interview questions, the data collectors probed them to get more detail and clear information.

### Variables and measurement

The availability of resources, including trained healthcare workers, was examined using 17 indicators, with weighted score of 35%. Compliance was used to assess HEWs’ adherence to the ICCM treatment guidelines by observing patient-provider interactions and conducting document reviews. We used 18 indicators and a weighted value of 40%.

Mothers’ /caregivers’/ acceptance of ICCM service was examined using 14 indicators and had a weighted score of 25%. The indicators were developed with a five-point Likert scale (1: strongly disagree, 2: disagree, 3: neutral, 4: agree and 5: strongly agree). The cut off point for this categorization was calculated using the demarcation threshold formula: ($$\frac{\text{t}\text{o}\text{t}\text{a}\text{l}\, \text{h}\text{i}\text{g}\text{h}\text{e}\text{s}\text{t}\, \text{s}\text{c}\text{o}\text{r}\text{e}-\,\text{t}\text{o}\text{t}\text{a}\text{l}\, \text{l}\text{o}\text{w}\text{e}\text{s}\text{t} \,\text{s}\text{c}\text{o}\text{r}\text{e}}{2}) +total lowest score$$(25–27). Those mothers/caregivers/ who scored above cut point (42) were considered as “satisfied”, otherwise “dissatisfied”. The indicators were adapted from the national ICCM and IMNCI implementation guideline and other related evaluations with the participation of stakeholders. Indicator weight was given by the stakeholders during EA. Indicators score was calculated using the formula$$\left(achieved \,in \%=\frac{indicator \,score \,x \,100}{indicator\, weight} \right)$$[26, 28].

The independent variables for the acceptability dimension were socio-demographic and economic variables (age, educational status, marital status, occupation of caregiver, family size, income level, and mode of transport), availability of prescribed drugs, waiting time, travel time to ICCM site, home to home visit, consultation time, appointment, and source of information.

The overall implementation of ICCM was measured by using 49 indicators over the three dimensions: availability (17 indicators), compliance (18 indicators) and acceptability (14 indicators).

#### Program logic model

**Fig. 1:**
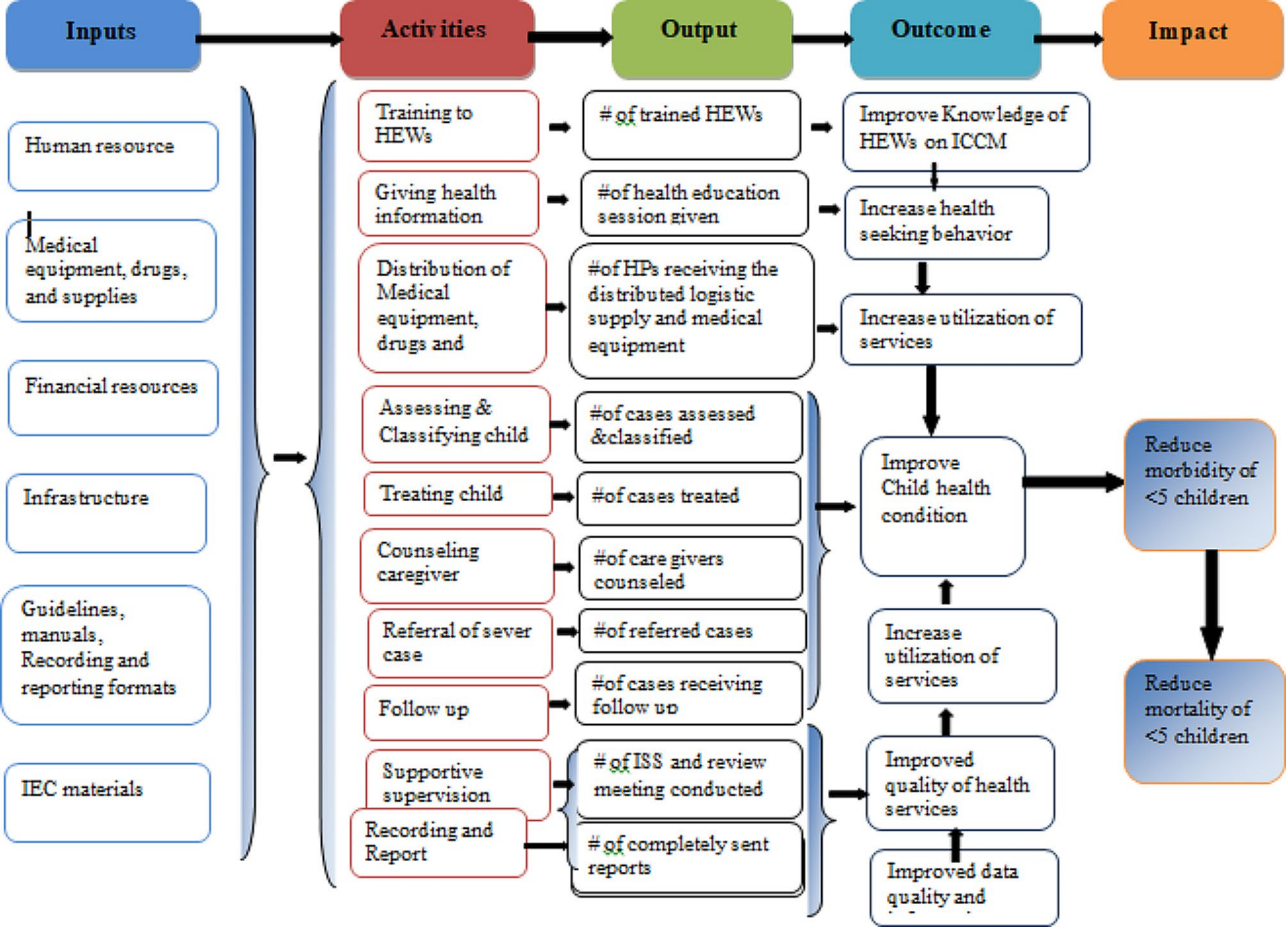
Integrated community case management of childhood illness program logic model in Gondar City in 2022

Based on the constructed program logic model and trained health care providers, mothers/caregivers received health information and counseling on child feeding; children were assessed, classified, and treated for disease, received follow-up; they were checked for vitamin A; and deworming and immunization status were the expected outputs of the program activities. Improved knowledge of HEWs on ICCM, increased health-seeking behavior, improved quality of health services, increased utilization of services, improved data quality and information use, and improved child health conditions are considered outcomes of the program. Reduction of under-five morbidity and mortality and improving quality of life in the society are the distant outcomes or impacts of the program (Fig. 1).

### Data collection tools and procedure

Resource inventory and data extraction checklists were adapted from standard ICCM tool and check lists [29]. A structured interviewer administered questionnaire was adapted by referring different literatures [30, 31] to measure the acceptability of ICCM. The key informant interview (KII) guide was also developed to explore the views of KIs. The interview questionnaire and guide were initially developed in English and translated into the local language (Amharic) and finally back to English to ensure consistency. All the interviews were done in the local language, Amharic.

Five trained clinical nurses and one BSC nurse were recruited from Gondar zuria and Wegera district as data collectors and supervisors, respectively. Two days training on the overall purpose of the evaluation and basic data collection procedures were provided prior to data collection. Then, both quantitative and qualitative data were gathered at the same time. The quantitative data were gathered from program documentation, charts of ICCM program visitors and, exit interview. Interviews with 21 KIIs and non-participatory observations of patient-provider interactions were used to acquire qualitative data. Key informant interviews were conducted to investigate the gaps and best practices in the implementation of the ICCM program.

A pretest was conducted to 26 mothers/caregivers/ at Maksegnit health post and appropriate modifications were made based on the pretest results. The data collectors were supervised and principal evaluator examined the completeness and consistency of the data on a daily basis.

### Data management and analysis

For analysis, quantitative data were entered into epi-data version 4.6 and exported to Stata 14 software for analysis. Narration and tabular statistics were used to present descriptive statistics. Based on established judgment criteria, the total program implementation was examined and interpreted as a mix of the availability, compliance, and acceptability dimensions. To investigate the factors associated with ICCM acceptance, a binary logistic regression analysis was performed. During bivariable analysis, variables with p-values less than 0.25 were included in multivariable analysis. Finally, variables having a p-value less than 0.05 and an adjusted odds ratio (AOR) with a 95% confidence interval (CI) were judged statistically significant. Qualitative data were collected recorded, transcribed into Amharic, then translated into English and finally coded and thematically analyzed.

### Judgment matrix analysis

The weighted values of availability, compliance, and acceptability dimensions were 35, 40, and 25 based on the stakeholder and investigator agreement on each indicator, respectively. The judgment parameters for each dimension and the overall implementation of the program were categorized as poor (< 60%), fair (60–74.9%), good (75-84.9%), and very good (85–100%).

## Results

### Availability of resources

A total of 26 HEWs were assigned within the fourteen health posts, and 72.7% of them were trained on ICCM to manage common childhood illnesses in under-five children. However, the training was given before four years, and they didn’t get even refreshment training about ICCM. The KII responses also supported that the shortage of HEWs at the HPs was the problem in implementing the program properly.

I am the only HEW in this health post and I have not been trained on ICCM program. So, this may compromise the quality of service and client satisfaction.(25 years old HEW with two years’ experience)

All observed health posts had ICCM registration books, monthly report and referral formats, functional thermometer, weighting scale and MUAC tape meter. However, timer and resuscitation bag was not available in all HPs. Most of the key informant finding showed that, in all HPs there was no shortage of guideline, registration book and recording tool; however, there was no OTP card in some health posts.

“Guideline, ICCM registration book for 2–59 months of age, and other different recording and reporting formats and booklet charts are available since September/2016. However, OTP card is not available in most HPs.”. (A 30 years male health center director)

Only one-fifth (21%) of HPs had a clean water source for drinking and washing of equipment. Most of Key-informant interview findings showed that the availability of infrastructures like water was not available in most HPs. Poor linkage between HPs, HCs, town health department, and local Kebele administer were the reason for unavailability.

Since there is no water for hand washing, or drinking, we obligated to bring water from our home for daily consumptions. This increases the burden for us in our daily activity. (35 years old HEW)

Most medicines, such as anti-malaria drugs with RDT, Quartem, Albendazole, Amoxicillin, vitamin A capsules, ORS, and gloves, were available in all the health posts. Drugs like zinc, paracetamol, TTC eye ointment, and folic acid were available in some HPs. However, cotrimoxazole and vitamin K capsules were stocked-out in all health posts for the last six months. The key informant also revealed that: “Vitamin K was not available starting from the beginning of this program and Cotrimoxazole was not available for the past one year and they told us they would avail it soon but still not availed. Some essential ICCM drugs like anti malaria drugs, De-worming, Amoxicillin, vitamin A capsules, ORS and medical supplies were also not available in HCs regularly.”(28 years’ Female PHCU focal)

The overall availability of resources for ICCM implementation was 84.2% which was good based on our presetting judgment parameter (Table 1).

**Table 1:** Summary of ICCM program resource availability indicators in Gondar city administration, 2022

Indicators	E	O	W	S	A	Judgment parameter
Number HP with trained health extension worker on ICCM/IMNCI	14	11	3.4	2.67	78.5	75–84.9 = Good
Number of HPs with no stock out of amoxicillin in the last six months	14	14	2.5	2.5	100	85–100 = very good
Number of HPs with no stock out of anti-malaria drugs in the last six months	14	14	2.5	2.5	100	85–100 = very good
Number of HPs with no stock out of zinc in the last six months	14	11	2	1.57	78.5	75–84.9 = Good
Number of HPs with no stock out of ORS in the last six months	14	14	2.5	2.5	100	85–100 = very good
Number of HP with no stock out Albendazole in the last six month	14	14	2.2	2.2	100	85–100 = very good
Number of HP with no stock out Vitamin A in the last six month	14	14	2.2	2.2	100	85–100 = very good
Number of HP with no stock out RUTF in the last six month	14	6	2.5	1.07	42.8	< 60 = poor
Number of HPS with no stock out of paracetamol in the last six months	14	12	2.4	2.05	85.4	85–100 = very good
Number of HP with MUAC measurement	14	14	1.6	1.6	100	85–100 = very good
Number of HP with functional thermometer	14	14	1.6	1.6	100	85–100 = very good
Number of HPS with functional weight scale	14	14	1.6	1.6	100	85–100 = very good
Number of HPs with ICCM guide line	14	14	2.7	2.7	100	85–100 = very good
Number of HPs having ICCM registration book	14	14	1.2	1.2	100	85–100 = very good
Number of HPs with monthly ICCM reporting format.	14	14	1	1	100	85–100 = very good
Number of HPS with functional ORT corner	14	3	2	0.42	21	< 60 = poor
Number of HPs with functional timer	14	0	1	0	0	< 60 = poor
Over all availability dimension			35	29.66	84.2	75–84.9 = Good

NB: E: expected, O: observed, W: weight, S: score ((observed X weight)/expected), A: achievement in percentage ((score/weight)*100)

### Health extension worker’s compliance

From the 42 patient-provider interactions, we found that 85.7%, 71.4%, 76.2%, and 95.2% of the children were checked for body temperature, weight, general danger signs, and immunization status respectively. Out of total (42) observation, 33(78.6%) of sick children were classified for their nutritional status. During observation time 29 (69.1%) of caregivers were counseled by HEWs on food, fluid and when to return back and 35 (83.3%) of children were appointed for next follow-up visit. Key informant interviews also affirmed that;

“Most of our health extension workers were trained on ICCM program guidelines but still there are problems on assessment classification and treatment of disease based on guidelines and standards this is mainly due to lack refreshment training on the program and lack of continuous supportive supervision from the respective body.” (27years’ Male health center head)

From 10 clients classified as having severe pneumonia cases, all of them were referred to a health center (with pre-referral treatment), and from those 57 pneumonia cases, 50 (87.7%) were treated at the HP with amoxicillin or cotrimoxazole. All children with severe diarrhea, very severe disease, and severe complicated malnutrition cases were referred to health centers with a pre-referral treatment for severe dehydration, very severe febrile disease, and severe complicated malnutrition, respectively. From those with some dehydration and no dehydration cases, (82.4%) and (86.8%) were treated at the HPs for some dehydration (ORS; plan B) and for no dehydration (ORS; plan A), respectively. Moreover, zinc sulfate was prescribed for 63 (90%) of under-five children with some dehydration or no dehydration. From 26 malaria cases and 32 severe uncomplicated malnutrition and moderate acute malnutrition cases, 20 (76.9%) and 25 (78.1%) were treated at the HPs, respectively. Of the total reviewed documents, 56 (93.3%), 66 (94.3%), 38 (84.4%), and 25 (78.1%) of them were given a follow-up date for pneumonia, diarrhea, malaria, and malnutrition, respectively.

Supportive supervision and performance review meetings were conducted only in 10 (71.4%) HPs, but all (100%) HPs sent timely reports to the next supervisory body.

Most of the key informants’ interview findings showed that supportive supervision was not conducted regularly and for all HPs.

I had mentored and supervised by supportive supervision teams who came to our health post at different times from health center, town health office and zonal health department. I received this integrated supervision from town health office irregularly, but every month from catchment health center and last integrated supportive supervision from HC was on January. The problem is the supervision was conducted for all programs.(32 years’ old and nine years experienced female HEW)

.

Moreover, the result showed that there was poor compliance of HEWs for the program mainly due to weak supportive supervision system of managerial and technical health workers. It was also supported by key informants as:

We conducted supportive supervision and performance review meeting at different time, but still there was not regular and not addressed all HPs. In addition to this the supervision and review meeting was conducted as integration of ICCM program with other services. The other problem is that most of the time we didn’t used checklist during supportive supervision. (Mid 30 years old male HC director)

Based on our observation and ICCM document review, 83.1% of the HEWs were complied with the ICCM guidelines and judged as fair (Table 2).

**Table 2:** Summary of compliance of HEWs to guidelines indicators in Gondar city, 2022

Indicators	E	O	W	S	A	Judgment parameter
Proportion of sick children who are asked for main problem/chief compliant	42	42	2.4	2.4	100.0	85–100 = very good
Proportion of sick children who are measured their weight	42	30	2.35	1.68	71.4	60-74.9 = fair
Proportion of sick children who are measured their temperature	42	33	2.35	1.84	78.3	75-84.9 = good
Proportion of sick children checked for danger sign according to ICCM guideline	42	32	2.7	2.06	76.3	75-84.9 = good
Proportion of children who are correctly assessed and classified for pneumonia, diarrhea, malaria and malnutrition according to ICCM guideline in the last three month.	230	230	4.5	4.5	100.0	85–100 = very good
Proportion of sick children with classification of pneumonia that are correctly treated according to ICCM guideline in the last three month.	70	63	2.4	2.16	90	85–100 = very good
Proportion of sick children with classification of diarrhea that are correctly treated according to ICCM guideline in the last three month.	82	72	2.4	2.11	87.9	85–100 = very good
Proportion of sick children with classification of malaria that are correctly treated according to ICCM guideline in the last three month.	54	48	2.4	2.13	88.7	85–100 = very good
Proportion of sick children with classification of malnutrition that are correctly treated according to ICCM guideline in the last three month	34	27	2.4	1.91	79.6	75-84.9 = good
Proportion of caregivers counseled about food, fluid and when to return according to ICCM guideline	42	29	2.25	1.55	68.9	60-74.9 = fair
Proportion of care givers appointed for follow up visit in the last three month.	230	208	2.25	2.03	90.2	85–100 = very good
Proportion of mothers and sick children checked for HIV/AIDS status according to ICCM guideline in the last three month.	230	171	1.75	1.3	74.3	60-74.9 = fair
Proportion of sick children checked for anemia according to ICCM guideline in the last six month.	230	153	1.75	1.16	66.3	60-74.9 = fair
Proportion of sick children checked for immunization status according to ICCM guideline in the last three month.	230	161	1.75	1.22	69.7	60-74.9 = fair
Proportion of sick children checked for vitamin A supplementation status according to ICCM guideline in the last three month.	218	179	1.75	1.43	81.7	75-84.9 = good
Proportion of sick children checked for de-worming status according to ICCM guideline in the last three month.	119	93	1.75	1.37	78.3	75-84.9 = good
Proportion of HPs supervised by higher health office in the last quarter.	14	10	1.65	1.18	71.5	75-84.9 = good
No of HPs sent report during reporting period.	14	14	1.2	1.2	100.0	85–100 = very good
Over all compliance dimension			40	33.2	83.1	75-84.9 = good

NB: E: expected, O: observed, W: weight, S: score ((observed X weight)/expected), A: achievement in percentage ((score/weight) X 100)

### Acceptability of ICCM program

#### Sociodemographic and obstetric characteristics of participants

A total of 484 study participants responded to the interviewer-administered questionnaire with a response rate of 95.3%. The mean age of study participants was 30.7 (SD ± 5.5) years. Of the total caregivers, the majority (38.6%) were categorized under the age group of 26–30 years. Among the total respondents, 89.3% were married, and regarding religion, the majorities (84.5%) were Orthodox Christian followers. Regarding educational status, over half of caregivers (52.1%) were illiterate (unable to read or write). Nearly two-thirds of the caregivers (62.6%) were housewives (Table 3).

**Table 3:** Socio-demographic characteristics of respondents for evaluation of ICCM in Gondar City, southern Ethiopia, 2022

Characteristics of caregivers	Category	Frequency (*n* = 484)	Percent
Age of caregivers	18–25	78	16.1
	26–30	187	38.6
	31–35	117	24.1
	>=36	102	21.1
Sex of caregivers	Male	34	7
	Female	450	93
Family size	< 4	168	34.7
	4–8	284	58.7
	9–12	32	6.6
Residence	Urban	69	14.3
	Rural	415	85.7
Income	<=500	41	8.5
	501–1000	183	37.8
	1001–2000	201	41.5
	2001–3000	50	10.3
	>300	9	1.9
Marital status	Married	423	89.3
	Single	12	2.5
	Widowed	27	5.6
	Divorce or separated	13	2.7
Religion of caregiver	Orthodox	409	84.5
	Muslim	68	14
	Protestant	7	1.5
Educational status	Unable to read and write	252	52.1
	Able to read and write	79	16.3
	Primary school	103	21.3
	Secondary school	36	7.4
	College and above	14	2.9
Occupational status	Government employee	22	4.5
	Farmers	99	20.5
	Trader/Merchants	40	8.3
	Housewife	303	62.6
	Daily labor	20	4.1
No of under five children	One	266	55
	Two	204	42.2
	Three	14	2.9

All the caregivers came to the health post on foot, and most of them 418 (86.4%) arrived within one hour. The majority of 452 (93.4%) caregivers responded that the waiting time to get the service was less than 30 min. Caregivers who got the prescribed drugs at the health post were 409 (84.5%). Most of the respondents, 429 (88.6%) and 438 (90.5%), received counseling services on providing extra fluid and feeding for their sick child and were given a follow-up date.

Most 298 (61.6%) of the caregivers were satisfied with the convenience of the working hours of HPs, and more than three-fourths (80.8%) were satisfied with the counseling services they received. Most of the respondents, 366 (75.6%), were satisfied with the appropriateness of waiting time and 431 (89%) with the appropriateness of consultation time. The majority (448 (92.6%) of caregivers were satisfied with the way of communicating with HEWs, and 269 (55.6%) were satisfied with the knowledge and competence of HEWs. Nearly half of the caregivers (240, or 49.6%) were satisfied with the availability of drugs at health posts.

The overall acceptability of the ICCM program was 75.3%, which was judged as good. A low proportion of acceptability was measured on the cleanliness of the health posts, the appropriateness of the waiting area, and the competence and knowledge of the HEWs. On the other hand, high proportion of acceptability was measured on appropriateness of waiting time, way of communication with HEWs, and the availability of drugs (Table 4).

**Table 4:** Summary of ICCM program acceptability by mothers in Gondar city, 2022

Indicators	E	O	W	S	A	Judgment parameter
Proportion of caregiver satisfied with convenience of working hour of health post? ( Availability of HEWs at working time)	484	298	1.2	0.74	61.7	60-74.9 = Fair
Proportion of caregiver satisfied with cleanness of the health post	484	255	1.1	0.58	52.7	< 60 = Poor
Proportion of caregiver satisfied with appropriateness of waiting area and comfortably of chair to receive ICCM service	484	219	1.25	0.57	45.6	< 60 = Poor
Proportion of caregiver satisfied with appropriateness of waiting time to receive ICCM service?	484	366	1.65	1.25	75.8	75–84.9 = Good
Proportion of caregiver satisfied with appropriateness of consultation time about ICCM service provided by HEW?	484	431	1.4	1.25	89.3	85–100 = Very good
Proportion of caregiver satisfied with counseling service about ICCM services that received from HEWs?	484	391	2.15	1.74	80.9	75–84.9 = Good
Proportion of caregiver satisfied with HEWs explanation about health status of child very well	484	362	1.8	1.35	75.0	75–84.9 = Good
Proportion of caregiver satisfied with the competence/Knowledge of the HEWs?	484	269	2.6	1.45	55.7	< 60 = Poor
Proportion of caregiver satisfied with the way of communication with HEWs? HEWs respect when receiving the service	484	448	2	1.85	92.5	85–100 = Very good
Proportion of caregiver satisfied with the availability of drugs at health post?	484	240	2.7	2.45	90.7	85–100 = Very good
Proportion of caregiver satisfied with the treatment medication given for the sick child by HEWs?	484	389	2.45	1.96	80.0	75–84.9 = Good
Proportion of caregiver agreed to recommend the service for other family or friends	484	341	1.25	0.88	70.4	60-74.9 = Fair
Proportion of caregivers having an interest to return back to health post by the next time to receive the service, if a child is sick?	484	409	1.45	1.22	84.1	75–84.9 = Good
Proportion of caregiver satisfied with the overall ICCM service provided.	484	374	2	1.54	77.0	75–84.9 = Good
Overall acceptability dimension			25	18.83	75.3	75–84.9 = Good

NB: E: expected, O: observed, W: weight, S: score ((observed X weight)/expected), A: achievement in percentage ((score/weight) X 100)

### Factors associated with acceptability of ICCM program

In the final multivariable logistic regression analysis, educational status of caregivers, availability of prescribed drugs, time to arrive, and waiting time were factors significantly associated with the satisfaction of caregivers with the ICCM program.

Accordingly, the odds of caregivers with primary education, secondary education, and college and above were 73% (AOR = 0.27, 95% CI: 0.11–0.52), 84% (AOR = 0.16, 95% CI: 0.07–0.39), and 92% (AOR = 0.08, 95% CI: 0.07–0.40) less likely to accept the program as compared to mothers or caregivers who were not able to read and write, respectively. The odds of caregivers or mothers who received prescribed drugs were 2.17 times more likely to accept the program as compared to their counters (AOR = 2.17, 95% CI: 1.14–4.10). The odds of caregivers or mothers who waited for services for less than 30 min were 2.8 times more likely to accept the program as compared to those who waited for more than 30 min (AOR = 2.80, 95% CI: 1.16–6.79). Moreover, the odds of caregivers/mothers who traveled an hour or less for service were 3.8 times more likely to accept the ICCM program as compared to their counters (AOR = 3.82, 95% CI:1.99–7.35) (Table 5).

**Table 5:** Bi-variable and multi-variable logistic regression analysis for factors affecting acceptability of ICCM program by caregivers/mothers/ in Gondar city, april 2022 (*n* = 484)

Variable	Category		COR (95% CI)	AOR(95% CI)
	Satisfied	Dissatisfied		
**Sex**				
Male	23	11	1	1
Female	346	104	0.46 (0.29 1.22)	1.54(0.61–3.91)
**Educational status**				
Illiterate	220	32	1	1
Able to read and write	63	16	0 0.57(0 0.29-1.11)	0.62(0.29–1.30)
Primary education	64	39	0 0.24(0.14–0.41)	0.27(0.12–0.52)*
Secondary education	19	17	0 0.16(0.07–0.34)	0.16(0.06–0.39)*
Collage and above	3	11	0.04(0.01–0.15)	0.08(0.07–0.39)*
**Occupational status**				
Government employee	8	14	1	1
Farmer	79	20	6.91(2.54–18.74)	2.11(0.61–7.39)
Trader/merchant	27	13	3.63(1.22–10.83)	1.87(0.47–7.40)
House wife	243	60	7.08(2.84–17.66)	2.41(0.74–7.74)
Daily laborer	12	8	2.62(0.75–9.13)	1.38(0.29–6.61)
**No of <5 children**				
One	210	56	2.08(0.67–6.46)	2.77(0.72–10.58)
Two	150	54	1.54(0.49–4.80)	2.02(0 0.53-7.66)
Three	9	5	1	1
**Counseling on food and fluid**				
Yes	338	91	2.87(1.60–5.14)	1.92(0 0.93-3.94)
No	31	24	1	1
**HEWs tell child illness**				
Yes	335	101	1.65(0.55–4.96)	1.85(0.73–4.69)
I don’t know	10	5	1.33(0.35–4.98)	1.06(0.24–4.63)
No	24	9	1	1
**Availability of prescribed drugs**				
Yes	325	84	2.72(1.62–4.57)	2.16(1.14–4.09)*
No	44	31	1	1
**Where the information got**				
From health professional	302	80	1.53(0.73–3.23)	0.98(0.45–2.11)
From family	27	11	0 0.679(0.28–1.612)	0.79(0 0.27-2.35)
From neighbor	40	24	1	1
**Arrive time**				
< 60 min	335	83	3.79(2.22–6.51)	3.82(1.98–7.35)*
*≥* 60 min	34	32	1	1
**Waiting time**				
< 30 min	351	101	2.70(1.29–5.62)	2.79(1.15–6.78)*
*≥* 30 min	18	14	1	1
**Consultation time**				
< 15 min	40	20	1(0.30–3.32)	0.74(0.16–3.34)
15–30 min	319	90	1.77(0.59–5.32)	1.46(0.36–5.84)
> 30 min	10	5	1	1
**Appointment given**				
Yes	344	94	3.07(1.64–5.73)	1.79(0.812–3.95)
No	25	21	1	1
**Home to home visit**				
Yes	354	97	4.38(2.13–9.01)	2.26(0 0.902 − 5.67)
No	15	18	1	1

* Significantly associated in multivariable logistic regression analysis at p-value < 0.05

### Overall ICCM program implementation and judgment

**Fig. 2:**
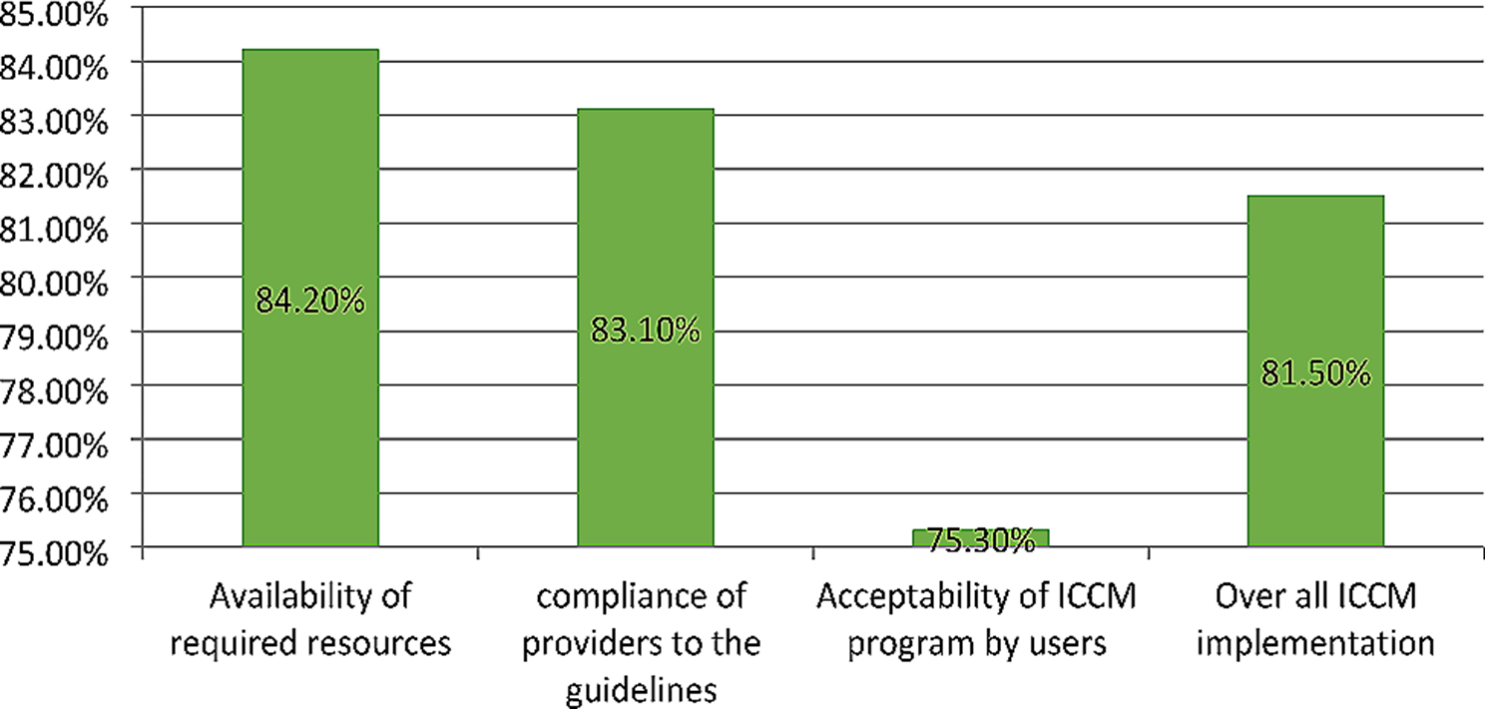
Overall ICCM program implementation and the evaluation dimensions in Gondar city administration, 2022

The implementation of the ICCM program in Gondar city administration was measured in terms of availability (84.2%), compliance (83.1%), and acceptability (75.3%) dimensions. In the availability dimension, amoxicillin, antimalarial drugs, albendazole, Vit. A, and ORS were available in all health posts, but only six HPs had Ready-to-Use Therapeutic Feedings, three HPs had ORT Corners, and none of the HPs had functional timers. In all health posts, the health extension workers asked the chief to complain, correctly assessed for pneumonia, diarrhea, malaria, and malnutrition, and sent reports based on the national schedule. However, only 70% of caretakers counseled about food, fluids, and when to return, 66% and 76% of the sick children were checked for anemia and other danger signs, respectively. The acceptability level of the program by caretakers and caretakers’/mothers’ educational status, waiting time to get the service and travel time ICCM sites were the factors affecting its acceptability. The overall ICCM program in Gondar city administration was 81.5% and judged as good (Fig. 2).

## Discussion

The implementation status of ICCM was judged by using three dimensions including availability, compliance and acceptability of the program. The judgment cut of points was determined during evaluability assessment (EA) along with the stakeholders. As a result, we found that the overall implementation status of ICCM program was good as per the presetting judgment parameter. Availability of resources for the program implementation, compliance of HEWs to the treatment guideline and acceptability of the program services by users were also judged as good as per the judgment parameter.

This evaluation showed that most medications, equipment and recording and reporting materials available. This finding was comparable with the standard ICCM treatment guide line [10]. On the other hand trained health care providers, some medications like Zink, Paracetamol and TTC eye ointment, folic acid and syringes were not found in some HPs. However the finding was higher than the study conducted in SNNPR on selected health posts [33] and a study conducted in Soro district, southern Ethiopia [24]. The possible reason might be due to low interruption of drugs at town health office or regional health department stores, regular supplies of essential drugs and good supply management and distribution of drug from health centers to health post.

The result of this evaluation showed that only one fourth of health posts had functional ORT Corner which was lower compared to the study conducted in SNNPR [34]. This might be due poor coverage of functional pipe water in the kebeles and the installation was not set at the beginning of health post construction as reported from one of ICCM program coordinator.

Compliance of HEWs to the treatment guidelines in this evaluation was higher than the study done in southern Ethiopia (65.6%) [24]. This might be due to availability of essential drugs educational level of HEWs and good utilization of ICCM guideline and chart booklet by HEWs. The observations showed most of the sick children were assessed for danger sign, weight, and temperature respectively. This finding is lower than the study conducted in Rwanda [35]. This difference might be due to lack of refreshment training and regular supportive supervision for HEWs. This also higher compared to the study done in three regions of Ethiopia indicates that 88%, 92% and 93% of children classified as per standard for Pneumonia, diarrhea and malaria respectively [36]. The reason for this difference may be due to the presence of medical equipment and supplies including RDT kit for malaria, and good educational level of HEWs.

Moreover most HPs received supportive supervision and performance review meeting was conducted and all of them send reports timely to next level. The finding of this evaluation was lower than the study conducted on implementation evaluation of ICCM program southern Ethiopia [24] and study done in three regions of Ethiopia (Amhara, Tigray and SNNPR) [37]. This difference might be due sample size variation.

The overall acceptability of the ICCM program was less than the presetting judgment parameter but slightly higher compared to the study in southern Ethiopia [24]. This might be due to presence of essential drugs for treating children, reasonable waiting and counseling time provided by HEWs, and smooth communication between HEWs and caregivers. In contrast, this was lower than similar studies conducted in Wakiso district, Uganda [38]. The reason for this might be due to contextual difference between the two countries, inappropriate waiting area to receive the service and poor cleanness of the HPs in our study area. Low acceptability of caregivers to ICCM service was observed in the appropriateness of waiting area, availability of drugs, cleanness of health post, and competence of HEWs while high level of caregiver’s acceptability was consultation time, counseling service they received, communication with HEWs, treatment given for their sick children and interest to return back for ICCM service.

Caregivers who achieved primary, secondary, and college and above were more likely accept the program services than those who were illiterate. This may more educated mothers know about their child health condition and expect quality service from healthcare providers which is more likely reduce the acceptability of the service. The finding is congruent with a study done on implementation evaluation of ICCM program in southern Ethiopia [24]. However, inconsistent with a study conducted in wakiso district in Uganda [38]. The possible reason for this might be due to contextual differences between the two countries. The ICCM program acceptability was high in caregivers who received all prescribed drugs than those did not. Caregivers those waited less than 30 min for service were more accepted ICCM services compared to those more than 30 minutes’ waiting time. This finding is similar compared with the study conducted on implementation evaluation of ICCM program in southern Ethiopia [24]. In contrary, the result was incongruent with a survey result conducted by Ethiopian public health institute in all regions and two administrative cities of Ethiopia [39]. This variation might be due to smaller sample size in our study the previous one. Moreover, caregivers who traveled to HPs less than 60 min were more likely accepted the program than who traveled more and the finding was similar with the study finding in Jimma zone [40].

### Strengths and limitations

This evaluation used three evaluation dimensions, mixed method and different data sources that would enhance the reliability and credibility of the findings. However, the study might have limitations like social desirability bias, recall bias and Hawthorne effect.

## Conclusion and recommendation

The implementation of the ICCM program in Gondar city administration was measured in terms of availability (84.2%), compliance (83.1%), and acceptability (75.3%) dimensions. In the availability dimension, amoxicillin, antimalarial drugs, albendazole, Vit. A, and ORS were available in all health posts, but only six HPs had Ready-to-Use Therapeutic Feedings, three HPs had ORT Corners, and none of the HPs had functional timers.

This evaluation assessed the implementation status of the ICCM program, focusing mainly on availability, compliance, and acceptability dimensions. The overall implementation status of the program was judged as good. The availability dimension is compromised due to stock-outs of chloroquine syrup, cotrimoxazole, and vitamin K and the inaccessibility of clean water supply in some health posts. Educational statuses of caregivers, availability of prescribed drugs at the HPs, time to arrive to HPs, and waiting time to receive the service were the factors associated with the acceptability of the ICCM program.

Therefore, continuous supportive supervision for health facilities, and refreshment training for HEW’s to maximize compliance are recommended. Materials and supplies shall be delivered directly to the health centers or health posts to solve the transportation problem. HEWs shall document the assessment findings and the services provided using the registration format to identify their gaps, limitations, and better performances. The health facilities and local administrations should construct clean water sources for health facilities. Furthermore, we recommend for future researchers and program evaluators to conduct longitudinal studies to know the causal relationship of the program interventions and the outcomes.

## Data Availability

Data will be available upon reasonable request from the corresponding author.

## Abbreviations

EDHS: Ethiopian Demographic and Health Survey
HC/HF: Health Center/Health Facility
HEP: Health Extension Program
HEW: Health Extension Workers
HP: Health Post
HSDP: Health Sector Development Plan
ICCM: Integrated Community Case Management of Common Childhood Illnesses
ICE: Information Communication and Education
IFHP: Integrated Family Health Program
IMNCI: Integrated Management of Neonatal and Childhood Illness
ISS: Integrated Supportive Supervision
MCH: Maternal and Child Health
MUAC: Mid Upper Arm Circumference
NGO: Non-Government Organization
ORS: Oral Rehydration Salts
OTP: Outpatient Therapeutic program
PHCU: Primary health care unit
RDT: Rapid Diagnostics Test
RUTF: Ready to Use Therapeutic Foods
SAM: Sever Acute Malnutrition
SNNPR: South Nation Nationalities People Region
UNICEF: United Nations International Child Emergency Fund
WHO: World Health Organization
